# Fluoropyrimidine sensitivity of human MCF-7 breast cancer cells stably transfected with human uridinehosphorylase

**DOI:** 10.1054/bjoc.2001.1833

**Published:** 2001-06

**Authors:** P Cuq, C Rouquet, A Evrard, J Ciccolini, L Vian, J-P Cano

**Affiliations:** Laboratoire de Toxicologie du Médicament, Faculté de Pharmacie, 15 av. Charles Flahault, Montpellier, cedex 02, 34060, France; Laboratoire de Toxicocinétique et Pharmacocinétique, 27 bd. Jean Moulin, Marseille cedex 5 13385, France

**Keywords:** uridine phosphorylase, breast cancer, 5-FU, 5′DFUR, cancer gene therapy

## Abstract

The relationship between uridine phosphorylase (UP) expression level in cancer cells and the tumour sensitivity to fluoropyrimidines is unclear. In this study, we found that UP overexpression by gene transfer, and the subsequent efficient metabolic activation of 5-fluorouracil (5-FU) by the ribonucleotide pathway, does not increase the fluoropyrimidine sensitivity of MCF-7 human cancer cells. © 2001 Cancer Research Campaign http://www.bjcancer.com


*British Journal of Cancer* (2001) **84**(12), 1677–1680
© 2001 Cancer Research Campaign
doi: 10.1054/ bjoc.2001.1833, available online at http://www.idealibrary.com on IDEAL®

http://www.bjcancer.com

### Fluoropyrimidine sensitivity of human MCF-7 breast cancer cells stably transfected with human uridine phosphorylase

**P Cuq[1,3], C Rouquet[1], A Evrard[1], J Ciccolini[2], L Vian[1] and J-P Cano[1]**

[1]Laboratoire de Toxicologie du Médicament, Faculté de Pharmacie, 15 av. Charles Flahault, 34060 Montpellier cedex 02, France; [2]Laboratoire de Toxicocinétique et Pharmacocinétique, 27 bd. Jean Moulin, 13385 Marseille cedex 5, France

**Summary** The relationship between uridine phosphorylase (UP) expression level in cancer cells and the tumour sensitivity to fluoropyrimidines is unclear. In this study, we found that UP overexpression by gene transfer, and the subsequent efficient metabolic activation of 5-fluorouracil (5-FU) by the ribonucleotide pathway, does not increase the fluoropyrimidine sensitivity of MCF-7 human cancer cells. © 2001 Cancer Research Campaign http://www.bjcancer.com

**Keywords**: uridine phosphorylase; breast cancer; 5-FU; 5′DFUR; cancer gene therapy

The pyrimidine nucleoside phosphorylases (PyNP), uridine (UP; EC 2.4.2.3) and thymidine (TP; EC 2.4.2.4) phosphorylase, are involved in the salvage pathway of pyrimidine nucleosides, but also in the activation of fluoropyrimidines. They are responsible for the conversion of the pro-drug 5′-deoxy-5-fluorouridine (5′-DFUR, doxifluridine) to its active form 5-fluorouracil (5-FU) (Armstrong and Diasio, 1980). Because of their reversible phosphorolytic activity, UP and TP also assume the conversion of 5-FU to its anabolites, respectively 5-fluorouridine (5-FUrd) and 5-fluoro-2′-deoxyuridine (5-FdUrd) (Ishitsuka et al, 1980; Peters et al, 1986).

PyNP gene transfer into cancer cells in order to increase the metabolic activation of fluoropyrimidines and thus the sensitivity of transfected cells to these anti-metabolites could be a new strategy in cancer gene therapy. Encouraging data have been obtained with TP gene transfer (Patterson et al, 1995; Schwartz et al, 1995; Kato et al, 1997; Evrard et al, 1999a, 1999b). Unlike TP, the data concerning UP are not numerous. NIH-3T3-derived cells expressing high levels of UP are more sensitive to fluoropyrimidines than parental cells (Watanabe et al, 1995). The increase of 5′DFUR antitumor activity by an extract from calf thymus (thymosin fraction 5) was attributed to an increase of UP activity (Ikemoto et al, 1999). In this paper, we studied the effects of UP overexpression by gene transfer in human breast cancer cells on the metabolic activation and on the cytotoxicity of fluoropyrimidines.

### MATERIALS AND METHODS

### Chemicals

5-FU, 5′-DFUR, enhanced chemoluminescence (ECL) and high-performance liquid chromatography (HPLC) reagents were from Sigma (St Quentin Fallavier, France). [$^3$H]5-FU (12.5 Ci mmol$^{-1}$) was from Du Pont de Nemours (Les Ulis, France).

### Cell lines

MCF-7 human breast adenocarcinoma cells (ATCC number HTB-22) were cultured at 37˚C in a fully humidified 5% $CO_2$ atmosphere in RPMI 1640 medium supplemented with 10% fetal calf serum (FCS) and 2 mM glutamine. COS-7 cells (ATCC number CRL-1651) were cultured in Dulbecco's Modified Eagle's Medium (DMEM) containing 5% FCS and 2 mM glutamine.

### UP overexpression in COS-7 and MCF-7 cells

The pUP-SG5 plasmid, containing the full length human UP cDNA (Watanabe and Uchida, 1995), was kindly provided by Dr SH Watanabe (Nippon Roche Research Center, Japan). The labelling of UP cDNA by a short *myc* sequence was performed by PCR using pUP-SG5, pFU polymerase (Stratagene, Amsterdam, The Netherlands) and specific primers. The *myc*/UP cDNA was ligated into the mammalian expression vector pcDNA3 (Invitrogen, NV Leek, The Netherlands) to produce pc*myc*/UP. Cells were transfected with pc*myc*/UP, pUP-SG5 or pcDNA3 (control) using Lipofectamine™ (Life Technologies, Cergy-Pontoise, France). COS-7 cells were lysed after 48 h incubation. Stable MCF-7 transfectants were selected for 250 µg ml$^{-1}$ geneticin (Life Technologies, Cergy-Pontoise, France) resistance.

### Immunoblotting

Proteins from cell lysates were electrophoresed on SDS-polyacrylamide gel, electroblotted onto nitrocellulose membrane, labelled with an anti-*myc* antibody (Invitrogen, France) and visualized using X-ray films after incubation with ECL reagents as previously described (Evrard et al, 1999b).

*Received 17 April 2000*
*Revised 13 March 2001*
*Accepted 15 March 2001*

*Correspondence to:* P Cuq

### UP activity assay

The UP activity was assayed as previously described (Watanabe and Uchida, 1995), with minor modifications. Cell lysates were incubated for 1 h at 37˚C in the presence of 10 mM uridine and the uracil, produced by uridine phosphorolysis, was quantified by HPLC as follows: C18 reverse phase column, 5 µm; mobile phase, $K_2$ $HPO_4$, 10 mM pH 4; flow rate, 0.5 ml $min^{-1}$; UV detection. The UP activity was expressed as nmol uracil mg $protein^{-1}$ $min^{-1}$.

### [$^3$H]5-FU metabolism analysis

After 4 h incubation in the presence of [$^3$H]5-FU (100 µCi), the tritiated metabolites in cell lysates were, as previously described (Ciccolini et al, 2000), separated by HPLC coupled to a radiometric detector (Flo-One Beta Radiomatic, Packard Instrument SA). Peaks were identified by comparing retention times with standards.

### In vitro cytotoxicity assay

The effects of antimetabolites on cell viability were assessed as previously described by using the neutral red assay (Evrard et al, 1996). The effect of the drugs on cell survival was expressed as percentage of viability of treated-cells compared with control cells.

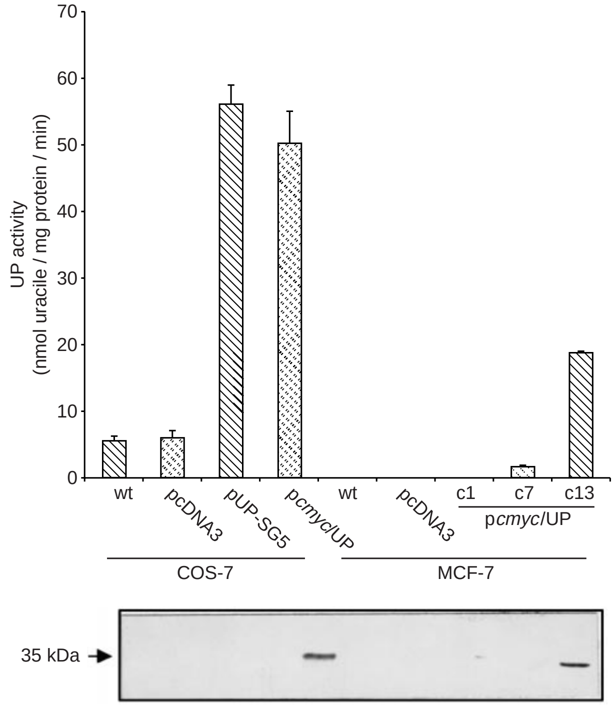

**Figure 1** UP expression and activity in COS-7 and MCF-7 cells. Upper panel: UP activity in lysates from parental (wt), pcDNA3-(negative control), pUP-SG5-(positive control), or pc*myc*/UP-transfected COS-7 cells, and from parental (wt), pcDNA3-or pc*myc*/UP-transfected MCF-7 cell clones (c1, c7 and c13). The uracil, resulting from uridine phosphorolysis by cell extracts, was quantified by HPLC. Data are means ± SEM of 3 separate experiments. Lower panel: proteins from COS-7 and MCF-7 cell lysates were electrophoresed by SDS-PAGE, blotted onto nitrocellulose sheets and labelled with an anti-*myc* antibody

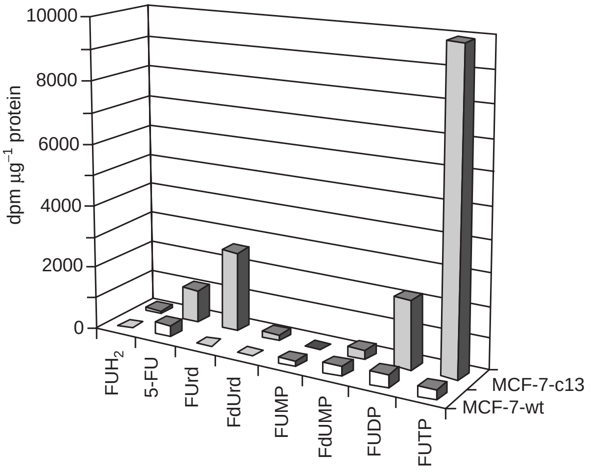

**Figure 2** Metabolism of [$^3$H]5-FU in MCF-7 cells. Parental or clone 13 MCF-7 cells were incubated for 4 hours in the presence of 100 µCi [$^3$H]5-FU (1.6 µM) and the resulting tritiated metabolites were identified by HPLC coupled to a radiometric detection. Data are from one representative experiment

### RESULTS

### Overexpression of UP in MCF-7 cells

No endogenous UP activity was detected in parental MCF-7 cells, as well as in pcDNA3-transfected cells (Figure 1). MCF-7 cells were transfected with the pc*myc*/UP plasmid which encoded a functional and immunodetectable *myc*-tagged UP, as assessed by transfection into COS-7 cells (Figure 1). Stable transfectants were selected for geneticin resistance. 3 clones (c1, c7 and c13) expressing different levels of UP were selected.

### [$^3$H]5-FU metabolism

Parental and clone 13 MCF-7 cells were incubated in the presence of [$^3$H]5-FU and the resulting metabolites were identified by HPLC coupled to a radiometric detection (Figure 2). [$^3$H]-fluorodeoxyuridine-monophosphate (FdUMP), and tritiated compounds of the RNA pathway (FUrd, FUMP, FUDP and FUTP) accumulated in parental MCF-7 cells. In MCF-7-c13 cells, the levels of the FUrd, FUDP and FUTP were strongly increased (about 89-, 5- and 38-fold, respectively).

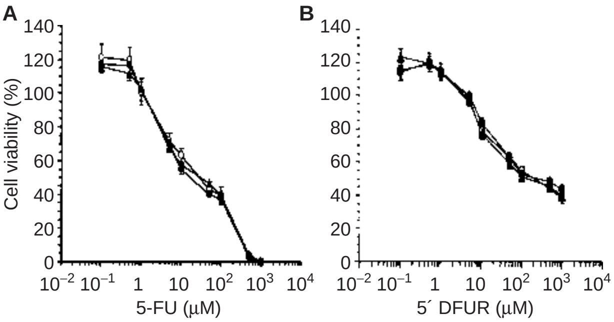

**Figure 3** Effect of UP expression on the in vitro sensitivity of MCF-7 cells to 5-FU and 5′DFUR. Parental (open circles) MCF-7-c7 (closed circles) or MCF-7-c13 (closed triangles) were incubated for 72 h at 37˚C in the presence of increasing concentrations (0.1 to 1000 µM) of 5-FU (**A**) and 5′DFUR (**B**). Cell viability was studied by using the neutral red uptake assay. Data, expressed as percentage of viability of treated cells compared with control cells, are means ± SEM of 4 separate experiments

*© 2001 Cancer Research Campaign*

### In vitro cytotoxicity

5-FU and 5′-DFUR decreased the viability of parental cells, as well as that of pc*myc*/UP-transfected cells (c7 and c13), in a concentration-dependent fashion (Figure 3). The fluoropyrimidine sensitivity of the *myc*/UP-overexpressing MCF-7 clones was not significantly (Student's *t*-test) different from that of parental cells, or from that of pcDNA3-transfected cells (data not shown).

### DISCUSSION

The metabolic activation of fluoropyrimidines can be initiated by UP and/or TP. Correlations between TP expression level and, on one hand the in vitro fluoropyrimidine cytotoxicity (Schwartz et al, 1995; Evrard et al, 1999a, 1999b), on the other hand the clinical outcome of fluoropyrimidine-based therapy (Koizumi et al, 1999; Saito et al, 1999) have been established. Thus, TP was proposed as a potential suicide gene for cancer gene therapy with 5′DFUR (Haraguchi et al, 1993; Patterson et al, 1995; Kanyama et al, 1999) or with 5-FU (Evrard et al, 1999a, 1999b). On the contrary, there was no clear demonstration of the importance of UP expression in tumours for fluoropyrimidine cytotoxicity. To address this question, UP was expressed by gene transfer in MCF-7 breast cancer cells. These cells were chosen because breast cancer is a major indication for 5-FU based therapy. Moreover, it was previously shown that MCF-7 cells do not express UP mRNA (Watanabe and Uchida, 1995); as expected, we did not detect any UP activity in parental cells.

HPLC experiments were performed to compare the [$^3$H]5-FU metabolic activation profiles in parental and MCF-7-c13 cells which exhibited the highest UP level. Parental cells displayed an accumulation of [$^3$H]FdUMP, reflecting the activation of [$^3$H]5-FU by the DNA pathway, but also of low amounts of 5-FU anabolites of the RNA pathway (FUrd, FUMP, FUDP and FUTP). These data were in agreement with previous reports (Kufe and Major, 1981; Peter et al, 1986; Patterson et al, 1995; Ackland and Peters, 1999) showing that MCF-7 cells can activate 5-FU through both RNA (initiated by TP) and DNA (initiated by FU conversion to FUMP by orotate phosphoribosyl transferase) pathways. When UP-expressing MCF-7-c13 cells were incubated in the presence of [$^3$H]5-FU, high levels of [$^3$H]FUrd and [$^3$H]fluororibonucleotides (FUDP, FUTP) were detected. This metabolic activation profile clearly indicates that human UP efficiently increases the metabolic activation of 5-FU through the RNA pathway when it is expressed in MCF-7 cells.

The effects of UP expression on the in vitro sensitivity of MCF-7 cells to antimetabolites were then studied. We found that MCF-7-c13 cells are not more sensitive to fluoropyrimidines than parental or pcDNA3-transfected cells. Thus, UP overexpression does not increase the 5-FU sensitivity of MCF-7 cells, even if the drug is effeciently metabolized. As reported by Patterson et al (1995), it seems likely that the DNA pathway is the most efficient activation pathway of fluoropyrimidines in MCF-7 cells. We previously obtained similar results (Evrard et al, 1999b) with LS174T colon cancer cells: despite an efficient metabolic activation of 5-FU by UP through the RNA pathway, parental LS174T cells were less sensitive to fluoropyrimidines than TP-transfected cells. Taken together, these data indicate that UP is not as important as TP for an efficient metabolic activation of fluoropyrimidines leading to a high cytotoxic effect.

In conclusion, this study shows that the UP activity level is not related to the sensitivity to 5-FU and 5′-DFUR of human MCF-7 breast cancer cells. Our data should provide rationale, on one hand for measuring TP rather than UP in clinical material in order to correlate with outcome of fluoropyrimidine based therapy, on the other hand for considering TP rather than UP as a potential suicide gene for cancer gene therapy with 5-FU or 5′DFUR.

### ACKNOWLEDGEMENTS

This work was supported by grants from Association pour la Recherche sur le Cancer, Ligue Nationale contre le Cancer (Comités de l'Ardèche et de l'Hérault) and Groupement des Entreprises Françaises dans la Lutte contre le Cancer.

### REFERENCES

Ackland SP and Peters GJ (1999) Thymidine phosphorylase: its role in sensitivity and resistance to anticancer drugs. *Drug Resist Update* **2**: 205–214

Armstrong RD and Diasio RB (1980) Metabolism and biological activity of 5′-deoxy-5-fluorouridine, a novel fluoropyrimidine. *Cancer Res* **40**: 3333–3338

Bradford MM (1976) A rapid and sensitive method for the quantitation of microgram quantities of protein utilising the principle of protein-dye binding. *Anal Biochem* **72**: 248–254

Ciccolini J, Peillard L, Evrard A, Cuq P, Aubert C, Pèlegrin A, Formento P, Milano G and Catalin J (2000) Enhanced antitumor activity of 5-fluorouracil in combination with 2′-deoxinosine in human colorectal cell lines and human colon tumor xenografts. *Clin Cancer Res* **6**: 1529–1535

Evrard A, Vian L, Aubert C and Cano JP (1996) An *in vitro* nucleoside analog screening method for cancer gene therapy. *Cell Biol Toxicol* **12**: 345–350

Evrard A, Cuq P, Robert B, Vian L, Pèlegrin A and Cano JP (1999a) Enhancement of 5-fluorouracil cytotoxicity by human thymidine phosphorylase expression in cancer cells: *in vitro* and *in vivo* study. *Int J Cancer* **80**: 465–470

Evrard A, Cuq P, Ciccolini J, Vian L and Cano JP (1999b) Increased cytotoxicity and bystander effect of 5-fluorouracil and 5-deoxy-5-fluorouridine in human colorectal cancer cells transfected with thymidine phosphorylase *Br J Cancer* **80**: 1726–1733

Haraguchi M, Furukawa T, Sumizawa T and Akiyama S (1993) Sensitivity of human KB cells expressing platelet-derived endithelial cell growth factor factor to pyrimidine antimetabolites. *Cancer Res* **53**: 5680–5682

Ikemoto S, Sugimura K, Wada S, Yasumoto R, Yamamoto K and Kishimoto T (1999) Augmentation of antitumor activity of 5′-deoxy-5-fluorouridine by thymosin fraction 5 in mouse bladder cancer cells in vitro and in vivo. *Cancer Lett* **145**: 121–126

Ishitsuka H, Miwa M, Takemoto K, Fukuoka K, Itoga A and Maruyama HB (1980) Role of uridine phosphorylase for antitumor activity of 5′-deoxy-5-fluorouridine. *Jpn J Cancer Res* **71**: 112–123

Kanyama H, Tomita N, Yamano T, Miyoshi Y, Ohue M, Fujiwara Y, Sekimoto M, Sakita I, Tamaki Y and Monden M (1999) Enhancement of the anti-tumor effect of 5′-deoxy-5-fluorouridine by transfection of thymidine phosphorylase gene into human colon cancer cells. *Jpn J Cancer Res* **90**: 454–459

Kato Y, Matsukawa S, Muraoka R and Tanigawa N (1997) Enhancement of drug sensitivity and a bystander effect in PC-9 cells transfected with a platelet-derived endothelial cell growth factor thymidine phosphorylase cDNA. *Br J Cancer* **75**: 506–511

Koizumi W, Saigenji K, Nakamaru N, Okayasu I and Kurihara M (1999) Prediction of response to 5′-deoxy-5-fluorouridine (5′-DFUR) in patients with inoperable advanced gastric cancer by immunostaining of thymidine phosphorylase/platelet-derived endothelial cell growth factor. *Oncology* **56**: 215–222

Kufe DW and Major PP (1981) 5-fluorouracil incorporation into human breast carcinoma RNA correlates with cytotoxicity. *J Biol Chem* **256**: 9802–9805

Patterson AV, Zhang H, Moghaddam A, Bicknell R, Talbot DC, Straford IJ and Harris AL (1995) Increased sensitivity to the prodrug 5′-deoxy-5-fluorouridine and modulation of 5-fluoro-2′-desoxyuridine sensitivity in MCF-7 cells transfected with thymidine phosphorylase. *Br J Cancer* **72**: 669–675

Peters GJ, Laurensse E, Leyva A, Lankelma J and Pinedo HM (1986) Sensitivity of human, murine, and rat cells to 5-fluorourcil and 5′-deoxy-5-fluorouridine in relation to drug-metabolizing enzymes. *Cancer Res* **46**: 20–28

© 2001 Cancer Research Campaign

Saito H, Tsujitani S, Oka S, Kondo A, Ikeguchi M, Maeta M and Kaibara N (1999) The expression of thymidine phosphorylase correlates with angiogenesis and the efficacy of chemotherapy using fluorouracil derivatives in advanced gastric carcinoma. *Br J Cancer* **81**: 484–489

Schwartz EL, Baptiste N, Wadler S and Makower D (1995) Thymidine phosphorylase mediates the sensitivity of human colon carcinoma cells to 5-fluorouracil. *J Biol Chem* **270**: 19073–19077

Watanabe SI and Uchida T (1995) Cloning and expression of human uridine phosphorylase. *Biochem Biophys Res Commun* **216**: 265–272

Watanabe SI, Hino A, Wada K, Eliason JF and Uchida T (1995) Purification, cloning, and expression of uridine phosphorylase. *J Biol Chem* **270**: 12191–12196

*© 2001 Cancer Research Campaign*

## References

[PDF_00307] Ackland Stephen P., Peters Godefridus J. (1999). Thymidine phosphorylase: its role in sensitivity and resistance to anticancer drugs.. Drug Resist Updat.

[PDF_00309] Armstrong R. D., Diasio R. B. (1980). Metabolism and biological activity of 5'-deoxy-5-fluorouridine, a novel fluoropyrimidine.. Cancer Res.

[PDF_00311] Bradford M. M. (1976). A rapid and sensitive method for the quantitation of microgram quantities of protein utilizing the principle of protein-dye binding.. Anal Biochem.

[PDF_00314] Ciccolini J., Peillard L., Evrard A., Cuq P., Aubert C., Pelegrin A., Formento P., Milano G., Catalin J. (2000). Enhanced antitumor activity of 5-fluorouracil in combination with 2'-deoxyinosine in human colorectal cell lines and human colon tumor xenografts.. Clin Cancer Res.

[PDF_00323] Evrard A., Cuq P., Ciccolini J., Vian L., Cano J. P. (1999). Increased cytotoxicity and bystander effect of 5-fluorouracil and 5-deoxy-5-fluorouridine in human colorectal cancer cells transfected with thymidine phosphorylase.. Br J Cancer.

[PDF_00320] Evrard A., Cuq P., Robert B., Vian L., Pèlegrin A., Cano J. P. (1999). Enhancement of 5-fluorouracil cytotoxicity by human thymidine-phosphorylase expression in cancer cells: in vitro and in vivo study.. Int J Cancer.

[PDF_00318] Evrard A., Vian L., Aubert C., Cano J. P. (1996). An in vitro nucleoside analog screening method for cancer gene therapy.. Cell Biol Toxicol.

[PDF_00327] Haraguchi M., Furukawa T., Sumizawa T., Akiyama S. (1993). Sensitivity of human KB cells expressing platelet-derived endothelial cell growth factor to pyrimidine antimetabolites.. Cancer Res.

[PDF_00330] Ikemoto S., Sugimura K., Wada S., Yasumoto R., Yamamoto K., Kishimoto T. (1999). Augmentation of antitumor activity of 5'-deoxy-5-fluorouridine by thymosin fraction 5 in mouse bladder cancer cells in vitro and in vivo.. Cancer Lett.

[PDF_00334] Ishitsuka H., Miwa M., Takemoto K., Fukuoka K., Itoga A., Maruyama H. B. (1980). Role of uridine phosphorylase for antitumor activity of 5'-deoxy-5-fluorouridine.. Gan.

[PDF_00337] Kanyama H., Tomita N., Yamano T., Miyoshi Y., Ohue M., Fujiwara Y., Sekimoto M., Sakita I., Tamaki Y., Monden M. (1999). Enhancement of the anti-tumor effect of 5'-deoxy-5-fluorouridine by transfection of thymidine phosphorylase gene into human colon cancer cells.. Jpn J Cancer Res.

[PDF_00341] Kato Y., Matsukawa S., Muraoka R., Tanigawa N. (1997). Enhancement of drug sensitivity and a bystander effect in PC-9 cells transfected with a platelet-derived endothelial cell growth factor thymidine phosphorylase cDNA.. Br J Cancer.

[PDF_00345] Koizumi W., Saigenji K., Nakamaru N., Okayasu I., Kurihara M. (1999). Prediction of response to 5'-deoxy-5-fluorouridine (5'-DFUR) in patients with inoperable advanced gastric cancer by immunostaining of thymidine phosphorylase/platelet-derived endothelial cell growth factor.. Oncology.

[PDF_00350] Kufe D. W., Major P. P. (1981). 5-Fluorouracil incorporation into human breast carcinoma RNA correlates with cytotoxicity.. J Biol Chem.

[PDF_00352] Patterson A. V., Zhang H., Moghaddam A., Bicknell R., Talbot D. C., Stratford I. J., Harris A. L. (1995). Increased sensitivity to the prodrug 5'-deoxy-5-fluorouridine and modulation of 5-fluoro-2'-deoxyuridine sensitivity in MCF-7 cells transfected with thymidine phosphorylase.. Br J Cancer.

[PDF_00357] Peters G. J., Laurensse E., Leyva A., Lankelma J., Pinedo H. M. (1986). Sensitivity of human, murine, and rat cells to 5-fluorouracil and 5'-deoxy-5-fluorouridine in relation to drug-metabolizing enzymes.. Cancer Res.

[PDF_00362] Saito H., Tsujitani S., Oka S., Kondo A., Ikeguchi M., Maeta M., Kaibara N. (1999). The expression of thymidine phosphorylase correlates with angiogenesis and the efficacy of chemotherapy using fluorouracil derivatives in advanced gastric carcinoma.. Br J Cancer.

[PDF_00366] Schwartz E. L., Baptiste N., Wadler S., Makower D. (1995). Thymidine phosphorylase mediates the sensitivity of human colon carcinoma cells to 5-fluorouracil.. J Biol Chem.

[PDF_00371] Watanabe S., Hino A., Wada K., Eliason J. F., Uchida T. (1995). Purification, cloning, and expression of murine uridine phosphorylase.. J Biol Chem.

[PDF_00369] Watanabe S., Uchida T. (1995). Cloning and expression of human uridine phosphorylase.. Biochem Biophys Res Commun.

